# Rationale and design of the HEALTHY-CATH trial: A randomised controlled trial of Heparin versus EthAnol Lock THerapY for the prevention of Catheter Associated infecTion in Haemodialysis patients

**DOI:** 10.1186/1471-2369-10-23

**Published:** 2009-08-20

**Authors:** Jennifer K Broom, Stacey O'Shea, Sridevi Govindarajulu, E Geoffrey Playford, Carmel M Hawley, Nicole M Isbel, Scott B Campbell, David W Mudge, Sally Carpenter, Barbara C Johnson, Neil P Underwood, David W Johnson

**Affiliations:** 1 Department of Medicine, Nambour General Hospital, Nambour, Australia; 2 Department of Nephrology, University of Queensland at Princess Alexandra Hospital, Brisbane, Australia; 3 Infection Management Service, University of Queensland at Princess Alexandra Hospital, Brisbane, Australia

## Abstract

**Background:**

Catheter-related bacteraemias (CRBs) contribute significantly to morbidity, mortality and health care costs in dialysis populations. Despite international guidelines recommending avoidance of catheters for haemodialysis access, hospital admissions for CRBs have doubled in the last decade. The primary aim of the study is to determine whether weekly instillation of 70% ethanol prevents CRBs compared with standard heparin saline.

**Methods/design:**

The study will follow a prospective, open-label, randomized controlled design. Inclusion criteria are adult patients with incident or prevalent tunneled intravenous dialysis catheters on three times weekly haemodialysis, with no current evidence of catheter infection and no personal, cultural or religious objection to ethanol use, who are on adequate contraception and are able to give informed consent. Patients will be randomized 1:1 to receive 3 mL of intravenous-grade 70% ethanol into each lumen of the catheter once a week and standard heparin locks for other dialysis days, or to receive heparin locks only. The primary outcome measure will be time to the first episode of CRB, which will be defined using standard objective criteria. Secondary outcomes will include adverse reactions, incidence of CRB caused by different pathogens, time to infection-related catheter removal, time to exit site infections and costs. Prospective power calculations indicate that the study will have 80% statistical power to detect a clinically significant increase in median infection-free survival from 200 days to 400 days if 56 patients are recruited into each arm.

**Discussion:**

This investigator-initiated study has been designed to provide evidence to help nephrologists reduce the incidence of CRBs in haemodialysis patients with tunnelled intravenous catheters.

**Trial Registration:**

Australian New Zealand Clinical Trials Registry Number: ACTRN12609000493246

## Background

Central venous catheterization is an increasingly common method of providing rapid, temporary access for the provision of haemodialysis (HD) to patients with serious acute or chronic kidney failure. Unfortunately, the clinical usefulness of this method is severely limited by the frequent occurrence of bloodstream infections in up to 40% of cases [1-4]. A number of registry [5,6] and observational cohort studies [7] have indicated that there has been an increasing reliance on haemodialysis catheters in incident haemodialysis patients ranging from 30% of patients in Europe and Australia to 60% in the United States of America [8-14]. Hospital admissions for catheter-related bacteraemias (CRBs) have doubled in the last decade [15] and a recent analysis of the Dialysis Morbidity and Mortality Wave 2 study [16] identified initial dialysis access as the main antecedent of bacteraemia in dialysis patients. Recent studies have further suggested that the use of haemodialysis catheters is associated with a 1.5- to 3-fold increase in both all-cause and infectious mortality [17-19].

Blood stream infections can result from extraluminal (exit site or tunnel infections) or intraluminal infection of the catheter [20]. Strategies for preventing CRBs have generally focused on cutaneous antisepsis, topical antibiotic application, antibiotic lock solutions and the use of devices coated or impregnated with antimicrobial agents [21,22]. However, these prophylactic strategies have been limited by concerns about antibiotic toxicity and the appearance of antibiotic-resistant microbial isolates [23,24]. Sodium citrate locks have also been investigated for prophylaxis against CRBs [25], although increased rates of catheter thrombosis have been reported [26].

Locking of catheters with ethanol is a promising technique in this respect as the agent is bactericidal, has low toxicity, is unlikely to produce resistant organisms, is able to disinfect organisms in biofilms, and is cheap. Ethanol is an antiseptic agent which is bacteriocidal by protein denaturation and is active against a wide variety of organisms including Gram-positive bacteria, Gram-negative bacteria, and fungi. In vitro studies have been published demonstrating the bactericidal effect of 70% ethanol against plastic-adherent organisms that commonly cause line infections [27]. Complete eradication of Gram-negative bacilli, Gram-positive cocci, and *Candida albicans *biofilms occurred with 30 minutes treatment with 60% ethanol compared to no eradication with trisodium citrate 46.7%.

The effects of catheter exposure to ethanol have been assessed and published. Silicone dialysis catheter integrity is maintained as assessed by scanning electron microscopy, even after 15 days exposure to 95% ethanol solutions [28]. The amount of silicone released was not significantly different when the catheter was submerged in ethanol compared to 0.9% sodium chloride. Mechanical testing of polyurethane and silicone catheters exposed to ten weeks of 70% ethanol proved ongoing structural integrity of both catheter types after prolonged ethanol exposure [29].

Published data has also demonstrated the effectiveness of ethanol lock therapy in the prevention of CRBs in haematology inpatients [16]. A short daily lock in haematology inpatients was used, which is not practical for long term use in renal dialysis outpatients. The current study is designed to ask whether a weekly lock could reduce the rate of CRBs in haemodialysis outpatients.

## Methods/design

Ethics approval for the trial has been obtained from the Princess Alexandra Hospital Human Research Ethics Committee prior to study initiation and patient enrolment. The study will be performed in accordance with the 2000 Edinburgh, Scotland Revision of the Declaration of Helsinki, the National Health and Medical Research Committee (NHMRC) Statement on Human Experimentation, Joint NHMRC/AVCC Statement and Guidelines on Research Practice, applicable ICH guidelines and the Therapeutic Goods Administration (TGA) – Note for guidance on good clinical practice (CPMP/ICH/135/95) annotated with TGA.

### Participants

**Figure 1:**
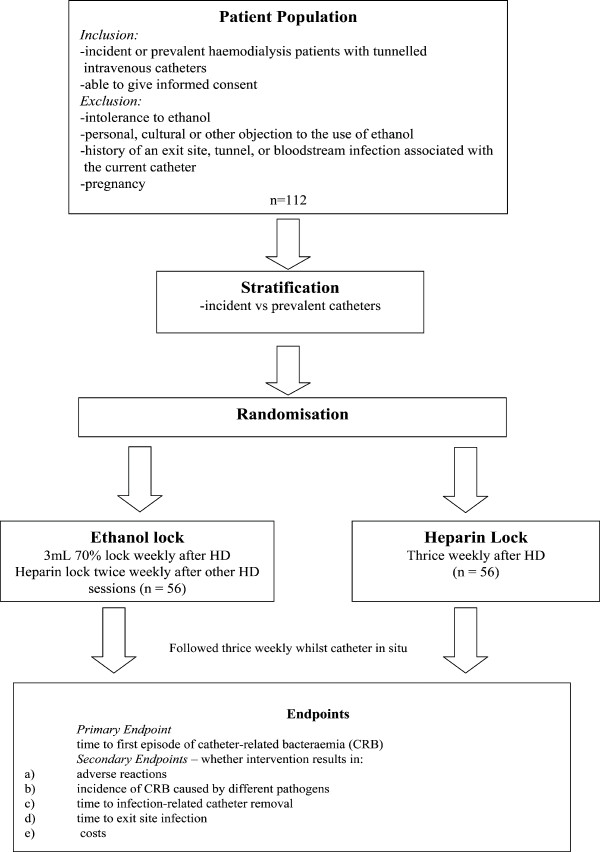
**Schema for the Ethanol Lock Trial**.

Participants will be selected from patients undergoing haemodialysis with incident and prevalent tunnelled intravenous catheters (Fig. 1). Inclusion criteria will include the presence of a tunnelled intravenous catheter and the ability to give informed consent. Exclusion criteria will include:

1. Pregnancy or breast-feeding

2. Religious or personal objection to the use of ethanol

3. Intolerance to ethanol

4. History of an exit site, tunnel or blood stream infection associated with the current catheter

### Study Design

The study will follow a prospective, open-label, randomised, controlled design. Adequate allocation concealment will be ensured using a centralised computer-generated block randomisation procedure. Randomisation will occur on the day that trial consent is obtained and will be conducted by calling a centralised number to allocate the patient to a trial arm. Stratification will occur for incident versus prevalent catheters.

### Experimental Intervention

Patients in the experimental arm will receive weekly instillation of 3 mL intravenous-grade 70% ethanol into each lumen of the catheter. Ethanol (Dehydrated alcohol USP injection, Phebra Pty Ltd, NSW 2066, Australia) is registered in Australia as an adjunct in the treatment of acute methanol and ethylene glycol poisoning and is supplied as a sterile solution of no less than 96.8% w/w of ethanol. Ethanol is diluted to 70% with sterile Water for Injections BP (Pfizer Australia Pty Ltd, NSW 2114 Australia) in a syringe prior to insertion into the catheter lumen. Dual lumen catheters receive 2 × 3 mL 70% ethanol, equivalent to 0.42 standard alcohol units. The ethanol lock is inserted immediately at the end of a haemodialysis session and left in situ until the next dialysis session (48 hours). When the patient returns for a further dialysis session, the ethanol lock will be aspirated out of each lumen of the catheter. For the remainder of the week patients in the experimental arm will receive standard heparin locks (Heparin sodium 5000 U/mL, Hospira, Germany).

### Control Intervention

Patients in the control arm will only receive the standard heparin lock into each lumen.

### Concurrent Treatments

Patients in each trial arm will undergo standard exit site and other management, as per the haemodialysis unit protocol. This will include thrice weekly exit site application of Medihoney (Medihoney™ Antibacterial Wound Gel, Medihoney Pty Ltd, Brisbane, Australia).

### Blinding

Blinding of investigators and patients is not possible as the distinctive odour of 70% ethanol is immediately recognisable and unable to be concealed. Outcome assessment based on standardised and objective criteria will be performed by persons blinded to treatment allocation.

### Outcome Measures

The primary outcome measure will be time to the first episode of CRB. CRB will be defined using standard objective criteria [30-32]: (a) probable catheter-related bacteraemia requires the isolation of a recognised pathogen from at least one set of blood cultures (or a potential skin contaminant from at least two sets of blood cultures) in the setting of clinical manifestations of sepsis with no other apparent source of sepsis other than the catheter; (b) definitive catheter-related bacteraemia requires the criteria of probable catheter-related bacteraemia and one of: purulence at the exit site; clinical sepsis that responds to antibiotic therapy upon catheter removal (after being refractory prior to catheter removal); or isolation of the same organism from the removed catheter tip.

Secondary outcomes will include:

a. Incidence of CRB caused by different pathogens

b. Time to exit site infection

c. Time to infection-related catheter removal

d. Time to catheter removal

e. Adverse reactions

f. Quality of life

g. Economic cost

### Clinical Assessment of Outcome

Reasons for removal of catheters will be assessed by medical staff. Blood cultures will only be taken as clinically indicated. The results of blood cultures collected during the study period will be recorded by research staff. Complications associated with lock therapy will be assessed on a weekly basis by nursing assessment. Patients will also be assessed for quality of life using the EQ-5D questionnaire, which will be administered by the study nurse on a monthly basis. They will also complete this questionnaire if admitted to hospital during their involvement in the trial. Data will be collected on health care service utilization external to the hospital (such as general practitioner visits) to allow economic evaluation in addition to the hospital-associated costs during the trial.

### Monitoring for Adverse Events

The number and proportion of subjections who report treatment-emergent adverse events will be summarised for each group.

A trial Safety and Data Monitoring Committee has been set up to allow external evaluation of catheter-related problems at 6 months and annually after that period. This will consist of Renal and Infectious Diseases Physicians not otherwise involved in the study, who will assess the significance of catheter-associated problems in the treatment vs control group.

### Sample Size Calculations

Prospective power calculations indicate that the study will have adequate statistical power (80% probability) to detect a clinically significant increase in infection-free survival from 200 days to 400 days if 56 patients are recruited into each arm (total 112 patients). These assumptions are based on local data in haemodialysis patients at the Princess Alexandra Hospital. A recruitment period of 3 years is anticipated.

### Statistical Analyses

Infection-free survival curves, survival probabilities and estimated survival times will be generated using Kaplan-Meier methodology. Data will be censored at the time of study completion and removal of catheter for non-infective reasons. The first occurrence of a relevant infective event will be considered an event. Differences in the survival curves will be assessed using the log rank test. All data will be analysed on an intention-to-treat basis. P values < 0.05 will be considered significant.

## Discussion

This trial has been designed to assess the efficacy of ethanol locks in the prevention of catheter-associated bacteremia in haemodialysis patients. Anecdotal and case series publications point to the potential efficacy of ethanol as a lock therapy in prevention and treatment of central venous catheter infection [33-35]. If effective, it will provide a cost-effective intervention which could substantially reduce patient morbidity and mortality, in addition to health-care associated costs. This trial will also prospectively assess adverse events that may be associated with ethanol use. In particular, the incidence of catheter-associated thrombosis has proven problematic in recent trials of sodium citrate locks [26]. There is a need for investigator-initiated trials of interventions such as this which do not attract pharmaceutical company funding or interest, but may provide important solutions to a clinical problems that confer substantial negative patient outcomes.

## Abbreviations

CKD: Chronic kidney disease; CRB: Catheter-related bacteraemia; ETOH: Ethanol; HD: Haemodialysis; HEALTHY-CATH: Heparin versus EthAnol Lock THerapY for the prevention of Catheter Associated infecTion in Haemodialysis patients; ICH: International Conference on Harmonisation; NHMRC: National Health and Medical Research Council; PAH: Princess Alexandra Hospital; TGA: Therapeutic Goods Administration.

## Competing interests

The authors declare that they have no competing interests.

## Authors' contributions

JB was the principal investigator; conceived study; participated in design and co-ordination; helped to draft manuscript; read and approved the final manuscript. SO participated in co-ordination; helped to draft manuscript; read and approved the final manuscript. SG participated in co-ordination; helped to draft manuscript; read and approved the final manuscript. GP participated in design and co-ordination; helped to draft manuscript; read and approved the final manuscript. CH participated in design and co-ordination; helped to draft manuscript; read and approved the final manuscript. NI participated in design and co-ordination; helped to draft manuscript; read and approved the final manuscript. SC participated in design and co-ordination; helped to draft manuscript; read and approved the final manuscript. DM participated in design and co-ordination; helped to draft manuscript; read and approved the final manuscript. DJ participated in design and co-ordination; helped to draft manuscript; read and approved the final manuscript.

## Pre-publication history

The pre-publication history for this paper can be accessed here:

http://www.biomedcentral.com/1471-2369/10/23/prepub
